# Development of a patient decision aid for people with refractory angina: protocol for a three-phase pilot study

**DOI:** 10.1186/1477-7525-12-93

**Published:** 2014-06-11

**Authors:** Michael Hugh McGillion, Sandra Lee Carroll, Kelly Metcalfe, Heather Mary Arthur, Joseph Charles Victor, Robert McKelvie, Etienne Marc Jolicoeur, Marie-Gabrielle Lessard, James Stone, Nelson Svorkdal, John George Hanlon, Ada Andrade, Joel Niznick, Louise Malysh, William McDonald, Bonnie Stevens, Peter Coyte, Dawn Stacey

**Affiliations:** 1 Faculty of Health Sciences, McMaster University, 1280 Main St. W, Hamilton ON, L8N 3Z5, Canada; 2 University of Toronto, Toronto M5T 1P8 ON, Canada; 3 General Division, Hamilton Health Sciences, 237 Barton Street East, Hamilton L8L, 2X2, ON, Canada; 4 Montreal Heart Institute, 5000, Bélanger Street, Montréal H1T 1C8 Québec, Canada; 5 Libin Cardiovascular Institute of Alberta, University of Calgary, C823, 1403 - 29th Street NW, Calgary T2N 2T9 AB, Canada; 6 Royal Jubilee Hospital, 1952 Bay Street, Victoria V8R 1J8, BC, Canada; 7 St. Michael’s Hospital, 30 Bond Street, Toronto M5B 1W8, ON, Canada; 8 Ottawa Cardiovascular Centre, Bank Street Professional Centre, 502-1355 Bank Street, Ottawa K1H 8K7, Canada; 9 St. Paul’s Hospital, 1081 Burrard Street, Vancouver V6Z 1Y6, BC, Canada; 10 School of Nursing, University of Ottawa, 451 Smyth Road, Ottawa K1H 8M5, ON, Canada

**Keywords:** Patient decision aid, Refractory angina, Pilot study, Study protocol

## Abstract

**Background:**

Refractory angina is a severe chronic disease, defined as angina which cannot be controlled by usual treatments for heart disease. This disease is frightening, debilitating, and difficult to manage. Many people suffering refractory have inadequate pain relief, continually revisit emergency departments for help, undergo repeated cardiac investigations, and struggle with obtaining appropriate care. There is no clear framework to help people understand the risks and benefits of available treatment options in Canada. Some treatments for refractory angina are invasive, while others are not covered by provincial health insurance plans. Effective care for refractory angina sufferers in Canada is critically underdeveloped; it is important that healthcare professionals and refractory angina sufferers alike understand the treatment options and their implications. This proposal builds on the recent Canadian practice guidelines for the management of refractory angina. We propose to develop a decision support tool in order to help people suffering from refractory angina make well-informed decisions about their healthcare and reduce their uncertainty about treatment options.

**Methods:**

This project will be conducted in three phases: a) development of the support tool with input from clinical experts, the Canadian refractory angina guidelines, and people living with refractory angina, b) pilot testing of the usability of the tool, and c) formal preliminary evaluation of the effectiveness of the support tool to help people make informed decisions about treatment options.

**Discussion:**

A decision support tool for refractory angina is needed and the available data suggest that by developing such a tool, we may be able to help refractory angina sufferers better understand their condition and the effectiveness of available treatment options (in their respective clinical settings) as well as their implications (e.g. risks vs. benefits). By virtue of this tool, we may also be able to facilitate identification and inclusion of patients’ values and preferences in the decision making process. This is particularly important as refractory angina is an intractable condition, necessitating that the selected course of treatment be lifelong. This study will yield a much needed patient decision aid for people living with refractory angina and pilot data to support a subsequent effectiveness study.

## Background

Refractory angina (RFA) is a debilitating chronic disease, resistant to conventional treatments for coronary artery disease (CAD) including nitrates, calcium-channel and β adrenoceptor blockade, vasculoprotective agents, percutaneous coronary interventions, and coronary artery bypass grafting [1,2]. Reasons for this habituation may include coronary anatomy that precludes effective repeat revascularizations, and extra-cardiac diseases that potentiate peri-operative morbidity (e.g. carotid stenosis, renal insufficiency) [2]. When untreated, repeated/chronic myocardial ischemia, inherent in RFA, can lead to maladaptive anaerobic glycolysis, tissue lactate production, accumulation of catabolites, and potassium efflux into the extracellular space [3,4]. Intrinsic compensatory mechanisms to correct these imbalances (e.g. myocardial hibernation, stunning) often fail, resulting in severe myocardial cellular dysfunction or death [3-5].

As more patients survive primary and subsequent cardiac events, the global prevalence of RFA is rising [2]. Estimates suggest that RFA affects between 600,000 and 1.8 million people in the United States [6] and that there are 30–50,000 new cases per year in continental Europe [2,6,7]. Canadian Community Health Survey data suggest that approximately 500,000 Canadians live with unresolved angina [8]. The annual mortality rate of patients living with RFA is not known but is thought to be in the range of approximately 3% [9]. Those living with RFA suffer severely impaired health-related quality of life (HRQL) including recurrent and sustained cardiac pain, psychological distress, impaired role functioning, activity restriction, and inability to self-manage [10,11].

In Canada, those with RFA struggle to obtain appropriate care. Some treatments for RFA are invasive, while others are not covered by provincial health insurance plans. Moreover, the effectiveness of these treatments varies by outcome [2,5]. To date, there is no clear, comprehensive patient decision making framework to help people understand the available treatment options and their relative risks and benefits.

### Patient decision aids

According to the International Patient Decision Aids Standard (IPDAS) collaboration [12,13] patient decision aids (PtDAs) are evidence-based tools designed to help people engage in deliberative treatment-related decision making by providing information on the options and outcomes relevant to health status. These tools are used to supplement, not replace, consultation between health care professionals (HCP) and patients [12,13]. In so doing, PtDAs help individuals to understand the range of options available and the probable consequences of various treatment options [12,13]. When designed optimally, PtDAs also distill for patients they value they place on the potential consequences of treatment approaches (e.g. invasive, non-invasive).

A recent (2011) Cochrane review of 86 studies [14] found that relative to routine health professional counseling, PtDAs improve patients’ knowledge of treatment options and perception of outcome probabilities, as well as their level of involvement in shared decision making. Ideally, effective PtDAs express outcome probabilities in both visual and written form to communicate absolute risk or benefit clearly to patients (i.e., X people out of 100) [15]. There is general consensus that decision support tools do not include passive informed consent materials, educational interventions not geared to a specific decision, or interventions designed to promote compliance with a recommended option rather than a choice on personal values [16,17]. Decision support tools have been developed in a range of formats including decision boards, interactive videodiscs, personal computers, audio-tapes, audio-guided workbooks, pamphlets, and group presentations [14].

### Study aims

The aims of this study are to:

1. Develop a decision support tool to promote and support informed RFA patient-health care professional decision making about available treatment options, and

2. Conduct preliminary testing of this tool.

Driven by the IPDAS quality framework [18] the specific aims of the PtDA are to:

1. Improve patients’ understanding of RFA, including the natural history of the condition if no action is taken,

2. Increase knowledge about the probable and relative effectiveness of available treatment options, as well as their potential risks and benefits, to solicit patients’ values and preferences about these treatment options, and to reduce RFA-treatment-related decisional conflict.

### Conceptual framework

The overarching conceptual framework giving direction to this project is the Ottawa Decision Support Framework [15]. Derived from concepts across general and social psychology, decision analysis, decision conflict, values, and social support and self-efficacy [19-25], this framework is an evidence-based, transdisciplinary, conceptual framework that can be used to guide the decision making process in clinical practice. The framework was developed for health decisions that a) are stimulated by a new circumstance, diagnosis, or developmental transition, b) require careful deliberation because of the uncertain and/or value-sensitive nature of the benefits and risks, and/or c) need relatively more effort during the deliberation phase than the implementation phase. The framework consists of three elements, including: assessment of needs or determinants of decisions, provision of decision support, and evaluation of the decision making process and outcomes of decisions made [15]. Evaluation of the quality of decision making is separated, conceptually, from the outcomes of care-related decisions themselves [15].

Hypotheses derived from the Ottawa Decision Support Framework [15], which have guided examination of the effects of decision support interventions, have been empirically tested. Decision support interventions, which include information, values clarification, and guidance in deliberation, have consistently produced hypothesized improvements in knowledge, treatment and outcome expectations, and decisional conflict in pre/post studies [26-30] as well as RCTs [14,31]. The available evidence, testing the propositions of the Ottawa Framework, appears to be consistent with the findings of the recent Cochrane review [14] on the overall effectiveness of decision support tools. On this basis, we concluded that the Ottawa Framework is a sound, theory-driven, and empirically supported conceptual framework to guide our development and preliminary evaluation of a PtDA for RFA.

## Methods

**Figure 1:**
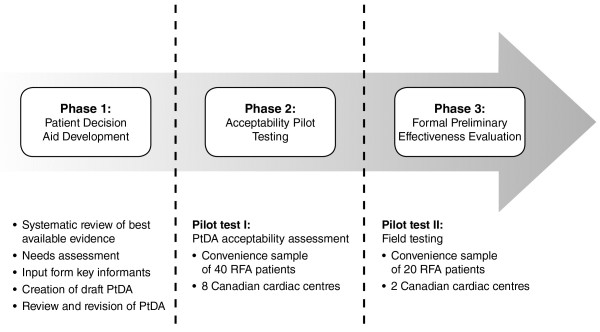
**Study flow diagram. ***PtDA patient decision aid; RFA refractory angina*.

To meet the research objectives, this project involves an overall three-phase PtDA development and pilot testing methodology (see Figure 1), adapted from previous, validated work [32]. The first phase, now underway, includes the development of the decision support tool and review by expert practitioners, and our key stakeholder interest groups including decision scientists, patient advocacy representatives, policy makers, and patients living with RFA. The second phase will involve pilot testing with a convenience sample, representative of people living with RFA from across Canada. The third phase will involve a local pretest-posttest evaluation of the decision support tool for people newly diagnosed with RFA. This study protocol was approved by the Hamilton Integrated Research Ethics Board (protocol reference [ref] #13-847), the University of Toronto Research Ethics Board (protocol ref #28970), the University Health Network Research Ethics Board (protocol ref #13-6667-AE), the Providence Health Care Research Ethics Board (protocol ref #H13-01094), and the Royal Jubilee Hospital Clinical Research Ethics Board (protocol re #C2014-022).

### Phase I: Development of the decision support tool

The steering committee overseeing this study is comprised of the investigative team, RFA patient representatives, our non-governmental organization decision making partners, clinician stakeholder representatives, and a web designer. This committee oversees all aspects of development and evaluation of the decision support tool. Driven by the Ottawa Decision Support Framework [15], the information, content, and format of the PtDA were developed via a) systematic review of the available evidence, b) steering committee input, c) evaluation of needs assessments of individuals with RFA, and d) input from all key informants, as follows:

#### Systematic review of the available evidence and data extraction

Multiple intervention approaches for RFA have been developed and tested (to varying degrees of rigour) in North America and continental Europe over the last two decades, including: neuromodulation techniques, enhanced external counter-pulsation, temporary cardiac sympathectomy, laser revascularization, high thoracic epidural anesthesia, heart rate modulating and metabolic agents, opioids, shock wave therapy, myocardial cryotherapy, coronary sinus reducer, stem cell therapy, and cognitive-behavioural interventions [5]. Prior to submission of this protocol for research ethics board (REB) approval, our investigative team developed and published the Joint Canadian Cardiovascular Society-Canadian Pain Society clinical practice guidelines (CPGs) for the management of RFA [5]. These CPGs featured a large-scale systematic review and meta-analysis of all above-listed treatment options for RFA; the evidence base included systematic reviews, randomized controlled trials (RCT), quasi-experimental studies, and pre-post observational designs. All studies were evaluated for methodological rigor using the Grading Recommendations, Assessment, Development and Evaluation (GRADE) system of evidence evaluation. Based on the IPDAS 2006 Checklist for Judging the Quality of Patient Decision Aids [33], we developed a data extraction form in order for the Research coordinator (RC) to extract the information needed on each treatment option for the PtDA (e.g. availability, safety, modes of action, logistical considerations, cost implications, and potential risks/benefits).

#### Needs assessment

We have published and are aware of several publications on the information and decision-related needs of people with RFA [10,11,34-36]. The RC abstracted relevant data from these publications including a) the natural course of RFA, b) patients’ experiences of various treatment options (e.g. life implications, side effects), c) degree of difficulty in making treatment-related choices, and d) attitudes about decision support.

#### Input from key informants

Input from key informants was obtained via a full-day, investigator-decision maker-collaborator consensus meeting in order to come to consensus on decision tool content and format. Our process was governed by an inductive consensus procedure entitled Technique for Research of Information by Animation of a Group of Experts (TRIAGE) [37]. Rooted in the constructivist paradigm of social research, the TRIAGE procedure differs from the Delphi Technique [38] and the Nominal Group Technique [38] in that exchange of ideas is not exclusively via control of a group leader or survey medium. Three formal steps are involved: 1) preparation, 2) individual production, and 3) interactive production [37]. Preparation: Our key informants included those directly involved in the governance of this research project as well as the primary authorship panel of the Joint RFA guidelines [5]. The RC mailed each participant a package containing a) a briefing document on our collective purpose, b) the Ottawa Decision Making Framework [15] and IPDAS quality checklist [33], c) the specific aims of the decision support tool, and d) a consensus conference worksheet. They were also sent an abridged version of the Joint RFA guidelines [5], highlighting the data on the effectiveness of the available treatment options, as well as outcome probabilities. Participants were asked to review these materials in advance of the conference. Individual production: Participants were asked to document up to 5 priorities for the PtDA on their worksheets, in light of the materials they reviewed. They then sent their worksheets back to the RC by mail or portable document format (PDF) via email. Prior to the consensus meeting, these priorities were categorized into the key ‘indicators’ or stems for discussion/debate. Interactive production: This final step involved the consensus conference itself. According to TRIAGE [37] a pre-established agenda is required as well as a facilitator who is competent in group dynamics management. An appointed facilitator guided the discussion to generate consensus on specific content around each ‘indicator’ (identified in Step 2). The mechanics of this process relied on a prominent visual aid, whereby ideas were documented as collectively ‘accepted’, ‘vetoed’, or ‘held’ for future consideration [37].

#### Creation of the draft decision aid

Information garnered from all preceding stages of Phase 1 was incorporated into the draft PtDA. The construction of the draft PtDA was based on the following components, according to the IPDAS quality checklist [33] framework and the guiding principles of the Ottawa Decision Framework [15], as follows:

##### A) Information about options and outcomes

The PtDA includes a description of the clinical situation (i.e., a diagnosis of RFA) that has stimulated the need to consider certain options and outcomes. Each treatment viable option was described in detail. The potential outcomes of each option were also described so that end-users will be able to understand what it may be like to experience each outcome. The functional impact of each outcome was also described (e.g. how the person can expect to respond to each treatment physically, emotionally, and socially) [15,18].

##### B) Presentation of probability of outcomes

One of the consistent benefits of PtDAs is to create realistic expectations of outcomes [14]. This was achieved by presenting probabilistic information about the likelihood of desirable and undesirable effects of all treatment options [15,18].

##### C) Values clarification

the PtDA was designed to ask participants to consider explicitly the personal importance of potential benefits and harms associated with each treatment option. The purpose of this valuing exercise was to structure and provide insight into how values affect personal decision making about options and to communicate those values [15,18]. This exercise was presented in the form of a ‘weigh scale’, wherein participants are presented with the potential desirable/undesirable effects of each option. They are then prompted to add any additional positive or negative factors which are important to them, check or shade each of these items in the ‘weigh scale’ to indicate their relative importance, and then indicate their predisposition toward one available treatment option versus others.

##### D) Coaching or Guidance

Guidance and coaching have been found to be helpful in promoting better coping strategies, health practices, and outcomes [14]. Structure and guidance in decision making was provided in the PtDA by illustrating the decision making process, including considering personal benefits and risks, b) clarifying level of certainty, knowledge and personal values, c) listing of current health practices, d) listing questions, e) indicating preferred role in decision making, and e) indicating current predisposition toward options. The general Ottawa Personal Decision Guide will was used to develop this illustrative process [39].

##### E) Delivery

For the purpose of this research study, the format of the PtDA for participants will be a paper-based version (see Phases 2 and 3). For our internal development and revision purposes, an electronic version of the tool has been housed on a secure, password-protected section of the website of one of our non-governmental organization partners.

#### Review and revisions to draft decision aid

The draft PtDA will be reviewed and critiqued by all stakeholders who were involved in our consensus conference proceedings, plus external RFA content experts. For the purposes of this review, we have created a secure, password-protected and interactive online environment entitled Decision Aid Manager. All reviewers will be asked to logon to Decision Aid Manager in order to view a PDF of the PtDA and assess its content using a quality assessment tool we adapted from the IPDAS quality checklist [33]. This exercise will include rigor of development as well as the effectiveness of the PtDA to help patients a) recognize that a decision needs to be made, b) know the available treatment options, as well as their features and potential risks and benefits (in equal detail), c) understand their values and preferences that affect decision making, and d) become involved in decision making in preferred ways. Decision Aid Manager includes both a ‘yes/no’ checklist for content-related items and a 5-point Likert scale for effectiveness-related items, ranging from 0 = ‘not effective at all’ to 4 = ‘very much effective’. This scale was adapted, with permission, from the effectiveness subscale of Sidani et al.’s Treatment Acceptability and Preference Questionnaire (TAP) [40]. The TAP has established internal consistency and validity, demonstrated by a 1-factor structure and significant differences in scores between patients with differing treatment preferences [40]. We will use our scale to assess areas of strengths and weaknesses of the PtDA; items rated as 2/4 (i.e. effective) or lower will indicate areas requiring attention. Our Decision Aid Manager also includes an open comment box to facilitate feedback on the overall presentation of the tool including clarity, user-friendliness, and visual appeal. Descriptive statistics will be computed by the biostatistician on our team and the RC will use the quantitative and qualitative data derived to create a feedback report of the findings of this review. As a form of integrated knowledge translation (KT), this report will be made available online to all reviewers via Decision Aid Manager, which also features a web-based discussion forum. We will host a week-long asynchronous discussion forum, calling for a collective ‘brainstorming’ session on how the PtDA should be revised, based on our feedback report. This study phase (i.e. review and feedback) will be carried out via a mail out and telephone-based feedback process for those who prefer paper-based media, or do not have convenient access to a computer.

### Phase 2: Pilot test 1

The purpose of Pilot 1 is to ensure that the PtDA is a) clearly formatted, b) acceptable to patients, and c) feasible for patients to complete (i.e. completion is with ease and time-efficient).

#### Sample and setting

Pilot 1 will involve a convenience sample of 40 RFA patients from across Canada. Those eligible will include patients who a) have a confirmed diagnosis of RFA [5], b) are currently being treated, and c) are able to read, speak and understand English. We are including experienced patients in this phase of the study because the effectiveness of the tool for supporting ‘good decisions’ cannot be presumed until accessibility is assessed; those newly diagnosed could be unduly influenced by a potentially unacceptable tool. This convenience sample will be recruited from 8 cardiac centres across Canada, with established cohorts of RFA patients undergoing active treatment.

Regional variations in RFA care exist across Canada with respect to availability of treatment options, requisite clinical skills, and insurance coverage [1]. Inherent in the notion of acceptability is relevance to our end-users. Hence, acceptability assessment by patients from across Canada is critical. Our total sample size of 40 patients is based on a review by Hertzog [41], suggesting a range of 20–40 participants to allow for sufficient variability in acceptability assessment of an intervention. Our acceptability data will be used to revise the PtDA, which will be tested as an intervention in a subsequent randomized controlled trial.

#### Procedure

The research process will be governed centrally. Each clinical site has a running list of known RFA patients. Designated third-party clinicians at each site will ask eligible patients if they would be willing to speak to the RC about the study. If verbal consent is obtained, contact information will be sent to the RC via telephone (to preserve confidentiality). The RC will then phone the referred individual to confirm eligibility, provide a detailed study explanation, and obtain preliminary verbal consent. Eligible patients agreeing to participate will be asked for baseline demographic information via phone interview. They will receive the study explanation, two copies of the consent form, and the draft decision support tool in the mail; a postage-paid, addressed envelope will be included to return one signed consent form to the RC. Participants will be asked to take 1–2 weeks to review the PtDA. The RC will schedule a follow-up phone call wherein participants will be asked to complete a PtDA Acceptability Questionnaire via phone interview; this process will take approximately 10–15 minutes. The RC will collate the data from this acceptability pilot and appropriate revisions will be made to the PtDA. A maximum of 5 participants per site is required. Based on up-front consultation with our clinical sites, we foresee no difficulty in obtaining the required sample.

### Phase 3: Pilot test 2 (pretest-posttest evaluation)

The overall aim of Pilot 2 is to field test the PtDA in the clinical setting. While the Cochrane review [14] of PtDAs demonstrated the general effectiveness of such tools for a) improving patients’ knowledge of the probable outcomes of various treatment options, b) changing patients initial treatment decisions/inclinations once relative risks were accurately perceived, and c) improving patients’ comfort and level of involvement with decision making, several questions remain. Clinical contextual factors may impact the effectiveness of a PtDA and will be taken into account, including the willingness of health care providers (HCPs) to use these tools, HCPs’ level of skill in shared decision making, and effectiveness of systems for decision support implementation. Operating in concert with such factors are the individual values and preferences which patients, and HCPs alike, bring to the clinical encounter. The degree to which these values and preferences are congruent with chosen treatment options varies [14]. Understanding these factors requires not only examination of PtDA effectiveness, but also the context of implementation and HCP-patient interactions during this process. We will therefore employ a combination of summative and formative research methods in Pilot 2.

While the Ottawa Decision Support Framework [15] guides our overall process for designing and testing the decision support tool, our conceptualization of Pilot-2 is also guided by the Ottawa Model of Research Use (OMRU) [42]; there are several elements of the OMRU that are conceptually consistent with the Ottawa Decision Support Framework. OMRU is an interactive model designed for the evaluation of KT innovation implementation. Within the OMRU [42] there are six key elements that interface to determine the process of knowledge translation: 1) evidence-based innovation, 2) potential adopters, 3) the practice environment, 4) implementation of interventions, 5) adoption of the innovation, and 6) outcomes of innovation implementation. In the context of Pilot-2, the innovation is the PtDA, the potential adopters are our participating HCPs and RFA patients, and the practice environment will constitute our Pilot 2 settings.

#### Primary purpose

The primary purpose is to determine the appropriateness and acceptability of the PtDA for our key effectiveness outcomes including decisional conflict (*primary outcome*) and knowledge of treatment options, and choice predisposition (*secondary outcomes*). This pilot test will allow us to determine the effect size of the PtDA, which will inform the required sample size for a randomized controlled trial (RCT) of the effectiveness of the tool as an intervention.

#### Secondary purpose

The secondary purpose is to a conduct a formative evaluation of the use of PtDA while in use. This evaluation will allow us to conduct a preliminary assessment of predisposing, enabling, and reinforcing factors [42] which may impact the ability of the tool to effectively translate knowledge about various treatment options, and support informed decision making, within the clinical context.

#### Sample and setting

Pilot 2 will involve a convenience sample of 20 RFA patients from two, large urban cardiac centres in Southern Ontario, Canada. Two centres will be used to allow for examination of differences in contextual factors that may impact the effectiveness of the PtDA. Those eligible will include patients who a) are diagnosed with RFA [15], b) require, and have not yet undergone, a specific treatment for RFA [15], c) have an appointment booked to speak with a site-designated HCP about treatment options within the study period, and d) are able to read, speak, and understand English. This sample size was chosen based on Hertzog’s recommendation [41] of a minimum of 20 participants for single sample pre-post pilot studies used to develop both estimates of effect size and variance for a RCT. larger trial. In addition, based on Cochrane data [14], we anticipate that for a level of significance of alpha = 0.05, power (1-beta) = 0.80, a standard deviation of 0.81, and a correlation between pretest and posttest scores of 0.80, we will be able to detect a difference of 0.34 in decisional conflict scores (range: 1–5). This represents a moderate effect size [43], which is typical of PtDAs [14] and also clinically meaningful given that it is has been observed between those who make decisions versus those who delay decisions [44].

As guided by OMRU [42], our implementation, structured adoption (for the purposes of pilot testing), and outcome evaluation will be as follows:

#### Field test procedure and outcomes

As an integrated KT strategy involving stakeholders from the inception of this project, Pilot 2 site co-investigators and collaborators are assisting us to establish site-designated HCPs, who will assist us with recruitment. These HCPs will be oriented to the study and procedures for pilot testing the PtDA during scheduled visits with their patients enrolled in the study. Potentially eligible patients, identified by these HCPs, will be asked if they would be willing to speak to the RC about the study. If verbal consent is obtained, contact information will be sent to the RC via telephone. The RC will then phone the referred individual to confirm eligibility, provide a detailed study explanation, and obtain preliminary verbal consent. Eligible patients agreeing to participate will be asked for baseline demographic information via phone interview. They will receive the study explanation, and two copies of the consent form; a postage-paid, addressed envelope will be included to return one signed consent form to the RC. Approximately 1 month prior to their scheduled visits with a site-designated HCP, the RC will conduct pre-test data collection via telephone interview, using our pre/post-test questionnaires. These instruments are designed to assess knowledge about treatment options, decisional conflict, and predisposition about treatment options. Upon completion of baseline data collection, the RC will mail participants a copy of the decision support tool. Participants will be asked to review the decision support tool and arrangements will be made for them to complete the PtDA Acceptability Questionnaire via telephone with the RC.

A paper-based version of the decision support tool will be implemented during participants’ scheduled visits with their site-designated HCPs. Where permission is granted (by participants and site-HCPs), the RC will make arrangements for these sessions to be audio-taped and later, transcribed verbatim. While discussing/deliberating treatment options, participants will be encouraged to share their thoughts on the decision support tool, ask questions about the available RFA treatment options, and discuss any concerns that they may have. This iterative approach supports the interactive principles of integrated knowledge translation [45-47]. One week following these scheduled visits, the RC will conduct post-test outcome assessment via telephone interviews, using our pre/post-test questionnaires.

#### Post-field test debriefing interviews

Once post-test measures are completed, the RC will conduct a brief, semi-structured follow up debriefing interview with each participant, focusing on the experience of using the decision support tool; this will also be conducted via telephone. These interviews will be conducted according to a semi-structured patient interview guide we have developed based on the OMRU knowledge translation framework. This guide will solicit participants’views about the impact of their values on decsions made, changes that may have occurred in treatment-related preferences and perceptions of risks/benefits, general reflections on the decision making process, applicability and usefulness of the decision support tool in context (e.g. barriers, facilitators to use), and their level of comfort interacting with the HCPs when using the tool. For efficiency of data collection, the RC will conduct a similar debriefing with our site HCPs in the form of focus groups; one focus group per site will be conducted. A semi-structured HCP focus group interview guide will also be used, also based on OMRU. These discussion questions will target HCPs’ views of the applicability and utility of the decsion support tool in their respective practice contexts, perceived efficacy of the tool in assisting their patients to arive at treatment-related decions, and perceived enablers and facilitators to decision tool implementation. The RC will take field notes, and these sesions will be audio-taped and transcribed verbatim, in preparation for qualitative content analyses.

#### Instrumentation: pre/posttest measures

##### Acceptability questionnaire

Decision support tool acceptability will be assessed using the Decision Aid Acceptability Questionnaire [32], comprised of open and closed-ended questions. Closed-ended questions will elicit feedback on amount of content, clarity, and helpfulness of the PtDA, acceptability of format, whether the information was presented in a balanced and fair manner, user satisfaction, and whether users would recommend it to other RFA patients. Evidence supports the validity of the Decision Aid Acceptability Questionnaire [32].

##### Decisional conflict scale

The Decisional Conflict Scale (DCS) [48] will be used to measure the primary effectiveness outcome of decisional conflict [49,50]. The purpose of this scale is to measure a person’s perception of difficulty in making a decision including, perceived uncertainty in choosing between options; modifiable factors contributing to uncertainty such as feeling uninformed, unclear about personal values, and unsupported; and quality of the choice selected. Choice quality is defined as choice that is informed, consistent with personal values, and personally satisfying satisfaction [32,48]. The DCS consists of 16 items. Scoring involves the summation and averaging of the items, ranging from 1 (low decisional conflict) to 5 (high decisional conflict). Scores of 2.0 or lower are associated with those who make decisions; scores of 2.5 or greater are associated with those who delay decisions [48]. The DCS has been used to assess health-related decisions across divergent health conditions and contexts [32,48]. Test-retest and internal consistency coefficients exceed 0.78. This scale varies in its ability to discriminate between different decision-supporting interventions [32,48].

##### Knowledge/comprehension test

Knowledge of RFA treatment options will be assessed using a knowledge questionnaire developed for this study. We will use a standardized, validated format developed by Metcalfe et al. [32] that includes items regarding the mechanisms of action and effectiveness of various (RFA) treatment options, as well as known possible adverse effects. Effectiveness estimates will be presented in the form of a continuous scale from 0 to 100%. Content knowledge items will be presented as ‘true’ or false’; these items will be developed as part of our consensus conference.

##### Choice predisposition tool

Participants’ decision predispositions will be measured using the Choice Predisposition Tool [15,32]. Participants will be asked to mark along a 15-point scale anchored by ‘not leaning towards’ or ‘leaning towards’ particular treatment options (e.g. Spinal cord stimulation, enhanced external counter-pulsation); a response option in the middle indicates ‘unsure’. Test-retest reliabilities of various iterations of this scale, across populations, exceed 0.90; they are also consistently correlated with personal values and expectations, and sensitive to change [15,32].

#### Data management and analyses

All electronic and audio-taped data will be secured in a locked facility. To inform sample size calculations and data analysis feasibility for a larger trial, data will be analyzed as in a larger study, and estimates of variance and correlation (i.e. intracluster correlation within site) will be generated. All quantitative data collected will be checked for departures from normality and analyzed initially with descriptive statistics; measures of central tendency and dispersion (e.g. means and standard deviations) will be computed for continuous variables. For categorical demographic variables, frequencies and proportions will be reported. Differences between pre and post-intervention measures will be examined using paired t-tests for all interval scaled measures, provided the assumption of normality is met [51]. Where the normality assumption is violated, the non-parametric Wilcoxon signed-rank test will be used [51].

As this protocol focuses on appropriateness, acceptability and pilot testing of the PtDA, no sub-analyses were planned to examine the influence of participant characteristics such as age, sex, and highest level of formal education on the outcomes of decisional conflict, knowledge of treatment items, and choice predisposition. Examination of the influence of participant characteristics will be undertaken in a subsequent effectiveness RCT.

An examination of missing data will be of particular importance to the pilot analysis. The level and pattern of missing data will be quantified. Presence versus absence of data for each scale will be compared across demographic and clinical characteristics to identify particular groups of individuals that may have a higher propensity for missing data.

All transcribed qualitative data, including needs assessment data, HCP-participant visits, participant follow-up interviews, and HCP focus groups will be analyzed using inductive thematic qualitative content analysis [52-54]. The frequency, extensiveness, and specificity of comments will guide data categorization into recurrent themes [52-54]. These themes will be altered and refined through a recursive process from the data to analyst-generated categorical and conceptual definitions.

## Discussion

This paper describes the protocol for the development and pilot testing of a PtDA for those who suffer RFA. A number of RCTs to date have examined the effectiveness of PtDAs in specific cardiac populations such as ischemic heart disease [55], newly diagnosed hypertension [56] and/or dyslipidemia [57], atrial fibrillation [58], and those referred for diagnostic coronary angiography [59]. The results of these primary trials corroborate with those of the 2011 Cochrane review [14]. Consistent improvements were found in patients’ knowledge of their conditions and available treatment options [56-59], decisional conflict and perceptions of potential outcome improvements and relative risks, and perceived levels of patient autonomy in the shared decision making process [55]. One RCT also found that engagement with a PtDA did not increase participants’ state anxiety [56], suggesting that informing cardiac patients about potential treatment risks, and addressing their preferences does not impose added psychological burden. In a RCT of a PtDA to augment pharmacist consultation about lipid lowering and/or antihypertensive pharmacotherapies, no impact on patients’ knowledge or risk perception was found [57]. However, clinician-patient interactions with the decision tool were brief and the complexity of the tool may have been a contributing factor [57].

While a PtDA has been designed specifically for angina [60] this tool targets therapies for chronic stable angina and unstable angina, two conditions which, unlike RFA, are amenable to standard medical therapies. Moreover, we found that this tool did not meet the internal quality and impact standards set forth by the IPDAS [18], receiving scores of 5/9 and 0/2 for development process and effectiveness criteria, respectively [33].

A decision support tool for RFA is needed and the available data suggest that by developing such a tool, we may be able to help RFA sufferers better understand their condition and the effectiveness of available treatment options (in their respective clinical settings) as well as their implications (e.g. risks vs. benefits). By virtue of this tool, we may also be able to facilitate identification and inclusion of patients’ values and preferences in the decision making process. This is particularly important as RFA is an intractable condition, necessitating that the selected course of treatment be lifelong [1,2,5,6].

This study will yield a much needed PtDA for people living with RFA and pilot data to support a subsequent effectiveness study.

### Study status

Phase 1 is complete and participants are currently being enrolled in Pilot 1.

## Abbreviations

CPG: Clinical practice guideline; DCS: Decisional conflict scale; ES: Effect size; GRADE: Grading recommendations, assessment, development and evaluation; HCP: Health care professional; IPDAS: International patient decision aids standard; KT: Knowledge translation; OMRU: Ottawa model of research use; PDF: Portable document format; PtDA: Patient decision aid; RC: Research coordinator; RCT: Randomized controlled trial; RFA: Refractory angina; TAP: Treatment acceptability and preference questionnaire; TRIAGE: Technique for research of information by animation of a group of experts.

## Competing interests

The authors of this protocol have no relevant financial conflicts or competing interests to declare.

## Authors’ contributions

MM participated in the design of this study, wrote the first draft of the manuscript, and applied for funding. MM, SLC, KM, HMA, JCV, RM, EMJ, JS, NS, PC, BS and DS participated in the design of this study, PtDA development, and critical review of this manuscript. All authors reviewed and approved the final manuscript.
